# Phenotyping of Drought-Stressed Poplar Saplings Using Exemplar-Based Data Generation and Leaf-Level Structural Analysis

**DOI:** 10.34133/plantphenomics.0205

**Published:** 2024-07-29

**Authors:** Lei Zhou, Huichun Zhang, Liming Bian, Ye Tian, Haopeng Zhou

**Affiliations:** ^1^ College of Mechanical and Electronic Engineering, Nanjing Forestry University, Nanjing 210037, P. R. China.; ^2^ Jiangsu Co-Innovation Center of Efficient Processing and Utilization of Forest Resources, Nanjing Forestry University, Nanjing 210037, P. R. China.; ^3^ State Key Laboratory of Tree Genetics and Breeding, Co-Innovation Center for Sustainable Forestry in Southern China, Key Laboratory of Forest Genetics & Biotechnology of Ministry of Education, Nanjing Forestry University, Nanjing 210037, P. R. China.; ^4^ College of Forestry and Grassland, Nanjing Forestry University, Nanjing 210037, P. R. China.

## Abstract

Drought stress is one of the main threats to poplar plant growth and has a negative impact on plant yield. Currently, high-throughput plant phenotyping has been widely studied as a rapid and nondestructive tool for analyzing the growth status of plants, such as water and nutrient content. In this study, a combination of computer vision and deep learning was used for drought-stressed poplar sapling phenotyping. Four varieties of poplar saplings were cultivated, and 5 different irrigation treatments were applied. Color images of the plant samples were captured for analysis. Two tasks, including leaf posture calculation and drought stress identification, were conducted. First, instance segmentation was used to extract the regions of the leaf, petiole, and midvein. A dataset augmentation method was created for reducing manual annotation costs. The horizontal angles of the fitted lines of the petiole and midvein were calculated for leaf posture digitization. Second, multitask learning models were proposed for simultaneously determining the stress level and poplar variety. The mean absolute errors of the angle calculations were 10.7° and 8.2° for the petiole and midvein, respectively. Drought stress increased the horizontal angle of leaves. Moreover, using raw images as the input, the multitask MobileNet achieved the highest accuracy (99% for variety identification and 76% for stress level classification), outperforming widely used single-task deep learning models (stress level classification accuracies of <70% on the prediction dataset). The plant phenotyping methods presented in this study could be further used for drought-stress-resistant poplar plant screening and precise irrigation decision-making.

## Introduction

Poplar (*Populus* L.) is a widespread fast-growing forest tree [1,2]. Poplar cultivation has received global attention because it is an important source of wood for industrial production and for the construction of protective forests [3–5]. Therefore, researchers have shown great interest in improving the woody biomass production of poplar. However, the productivity of poplar forests is limited by abiotic and biotic stresses [6]. Drought stress (or water deficit) is a severe abiotic stress that negatively affects material transport in plants and weakens photosynthesis, ultimately leading to a decrease in production [7,8]. These plant stress factors prompt us to explore innovative technologies for ensuring the growth and ultimate yield of plants. To overcome the challenges of drought stress in poplar plants, there are 2 typical solutions: improving plant management (e.g., water deficiency plant detection) and improving plants (e.g., cultivation of drought-resistant poplar varieties) [9,10]. Under traditional planting methods, the discrimination of water-deficient plants or the screening of drought-resistant varieties rely on manual monitoring. The low efficiency and poor accuracy of these methods limit their application.

The phenotype, also known as the performance of a plant, is determined by the genotype, growth environment, and their interactions [11], which can reflect the structural and functional characteristics of plant cells, tissues, organs, plants, and populations. Under drought stress, plants may exhibit abnormal phenotypic characteristics, such as wilting, curling, and changes in color and other physical and chemical indicators. Therefore, plant phenotyping technology has been widely studied for determining the growth status of plants. Determination of the water supply or water status of plants based on phenotypic changes could be further used for irrigation decision-making and plant drought tolerance evaluation.

Recently, spectral analysis [12,13], machine vision [14], laser scanning [15], and artificial intelligence technologies [16] have received widespread attention in the field of plant science, leading to the emergence of high-throughput plant phenotyping technology. This approach provides new solutions for plant drought stress analysis [17–19]. Digital sensors capture the morphological, physiological, and biochemical traits of drought-stressed plants, and intelligent data processing algorithms extract abnormal information related to plant drought stress. For instance, spectroscopic diagnostic technologies determine plant water status by analyzing absorption or reflectivity at different wavelengths [20,21]. Wong et al. [22] predicted the water content of bean plants in outdoor fields using hyperspectral remote sensing, achieving *R*^2^ values of 0.20 to 0.55, and these methods were further used for analyzing the drought response of common and tepary beans. Zhang et al. [23] conducted tomato leaf moisture determination using terahertz spectroscopy, realizing an *R*^2^ value of 0.972 and a root mean square error of 0.053. In these cases, satisfactory performances were obtained; however, the sensing devices and systems used are expensive and require much space to house. Computer vision could be considered an alternative low-cost technology. Plants undergoing drought stress were identified using computer vision based on their morphological and color characteristics [24]. Some of the most important water-content-related morphological traits, leaf angle and leaf wilting, can be calculated by plant image processing; however, fully automated implementation of this process is difficult [25]. There have been some published cases in which plants in images were directly classified as having different drought levels, but the compatibility of methods considering different varieties and different degrees of drought still needs to be studied [26,27]. Currently, deep learning algorithms are among the most popular intelligent information processing tools [28–30], making it possible for us to extract more useful information from color images. They are adopted to establish end-to-end predictive models that correlate the input image and the target output, such as plant variety, plant stress degree, the position of different components, and plant disease level. However, the large number of annotated samples required to train high-performance deep learning models for plant phenotyping cannot be ignored. Generative adversarial networks can be used for generating realistic plant images and have achieved success in plant leaf counting tasks [31]. However, it is difficult to generate the corresponding ground truth for more complex tasks, such as leaf segmentation. Moreover, most of the existing studies have not explored the influence of plant variety on plant drought stress detection models. The answer to the question “is the captured response signal derived from the differences in variety or from drought stress?” remains unclear. There is still much room to explore in regard to the detection of poplar drought stress and the phenotyping of stressed poplar plants.

In this study, a combination of computer vision and deep learning was used for poplar sapling phenotyping and poplar drought stress grading. The main contributions of this study are summarized as follows: (a) An exemplar-based synthetic poplar plant image generation method was proposed for dataset augmentation, reducing the number of manual image annotations involved in poplar leaf segmentation model training. (b) An automatic analysis method for leaf-level structural digitization and angle calculation based on segmented leaves was developed for detecting the response of plants to drought stress. (c) Multitask learning models were utilized to simultaneously discriminate the variety and level of drought stress experienced by poplar saplings, improving the performance of the stress detection models.

## Materials and Methods

### Poplar sapling samples

Four varieties of poplar saplings with different drought resistance abilities were selected for the experiment, namely, Siyang-1 poplar (SY1), 3804, 895, and 110. All the saplings were cultivated in an experimental field located at Baguazhou, Nanjing, Jiangsu, China. The plants were covered by a transparent plastic shed with a height of 3 m, preventing the impact of natural rainfall on the experiments. For each variety, 72 plants were selected for analysis, for a total of 288 plants. All these samples were supported using the same cultivation mode, with adequate irrigation and an appropriate amount of pesticides for disease and pest control. Before drought stress was implemented, all plants were watered every 3 d. After 70 d (21 June 2023), different levels of drought stress were applied to these plants. The plants were divided into 5 groups: control check (CK), moderate drought (MD), severe drought (SD), SD and normal rewatering (NW), and SD and moderate rewatering (MW). The CK group was maintained at the previous irrigation frequency and was watered every 3 d. The plants in the MD group were watered every 6 d. The plants in the SD group were not watered until death. For the NW group, irrigation was stopped until the poplar saplings showed obvious symptoms of leaf yellowing and wilting, after which the plants were watered every 3 d. For the MW group, a similar process to that used for the NW group was applied, but the watering frequency was once every 6 d after resuming irrigation. The overall introduction of the experimental samples is summarized in Table 1.

**Table 1.** Profile of the analyzed poplar sapling samples

Variety	Drought stress level^a^				
	CK	NW	SD	MW	MD
SY1	16	16	8	16	16
3804	16	16	8	16	16
895	16	16	8	16	16
110	16	16	8	16	16

^a^For the SD stress level, only 8 samples of each variety were collected. The other 8 samples were completely destroyed by drought stress.

### Data collection

A camera, ZED Mini 2 (Stereolabs, USA), was used to capture the red–green–blue (RGB) images of the studied poplar plant samples. The image resolution was set to 1,920 × 1,080. A black cloth with high absorbance was used as the imaging background. Images of the 288 selected plants were captured at 2 time points (at 3 and 4 weeks after the plants were subjected to drought stress). In total, 576 plant images of different varieties and under different stress levels were obtained.

### Image annotation preparation method

**Fig. 1.**
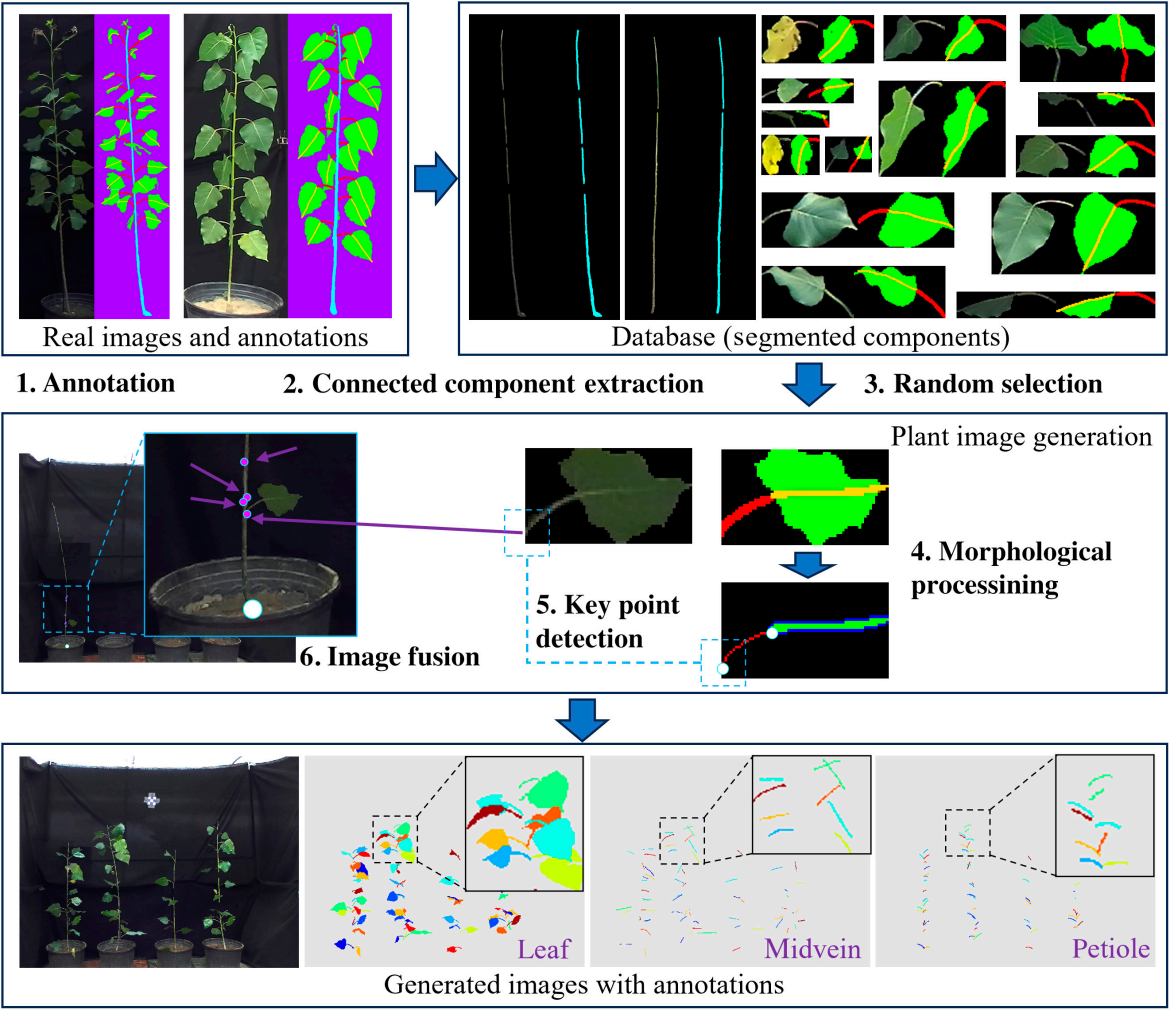
The proposed poplar plant image generation method.

This study focused on reducing manual labeling costs for deep learning applications. A novel dataset augmentation method was created for automatically generating poplar plant images with annotations. The included steps are shown in Fig. 1.

First, a small number of real images (32 individual plants were used) were manually annotated using the free image processing software Paint.net [32]. The regions of interest (ROIs) of each individual leaf, as well as the included regions of the midvein and the petiole, were annotated using green, yellow, and red, respectively. An example can be found in step 1 in Fig. 1. The annotated region of each individual leaf had to be separated, which was convenient for the subsequent “component segmentation” step. Step 2 included only a simple connected component extraction method. After finishing step 2, a database was constructed, covering subimages of leaves (350 images), trunks (32 images), and the corresponding annotations. Next, the leaves in the “database” were randomly selected to construct a simulated plant; see steps 3 to 6 illustrated in Fig. 1. Since each of the components in the “database” had been annotated with ROIs, the simulated plant images could be generated together with their annotations. Therefore, manual annotation could be avoided. An example of a generated plant image and the corresponding annotation maps is also shown in Fig. 1. The generated plant image looks very similar to the real captured images. It should be noted that the annotations of this type are compatible with the MaskRCNN series [33] segmentation methods. The YOLO series [34,35] segmentation methods require an annotation file with an “.txt” extension, in which each row contains the object class index and object bounding coordinates; refer to https://docs.ultralytics.com/datasets/segment/. An annotation format conversion algorithm (MaskRCNN format to YOLO format) was created on the basis of edge point extraction. The codes for conducting the proposed poplar plant image generation method and for annotation format conversion were uploaded to the GitHub platform (https://github.com/L-Zhou17/Plant-Image-Generation).

### Deep learning models

In this research, deep learning methods, including key phenotypic parameter measurements and drought stress level determination, were used for poplar saplings.

#### Instance segmentation for leaves

For the first objective, key component extraction (petiole, midvein, and leaf) had to be completed first. Instance segmentation [36] was the best choice. Therefore, 2 kinds of typical instance segmentation models, namely, MaskRCNN [33] and YOLOv8 segmentation [37], were adopted to extract the petiole, midvein, and leaf regions. Then, morphological parameters directly related to plant water status, including the angle of the petiole and that of the midvein, could be calculated. On the basis of the extracted components and the calculated key parameters, digital representation of the plant structure could be achieved.

For training and optimizing the MaskRCNN and YOLOv8 segmentation models, only the generated plant images obtained using the method described in the “Deep learning models” section were used. In total, 600 images were generated, 400 of which were used for training, and the remaining 200 images were used for validation. Then, 60 plants of different varieties and with different degrees of stress were manually annotated for prediction. Therefore, the proposed method used simulated images with annotations for training and real plant images for prediction and model performance evaluation. The initial weights and biases of the segmentation models were pretrained on the COCO dataset. The learning rate was 0.0005. The performance indicator AP_0.5_ (average precision using 0.5 as the threshold for intersection over union), which is widely used in deep learning instance segmentation applications, was calculated in this study. The model with the highest validation AP_0.5_ value was saved as the best model.

#### Image classification

**Fig. 2.**
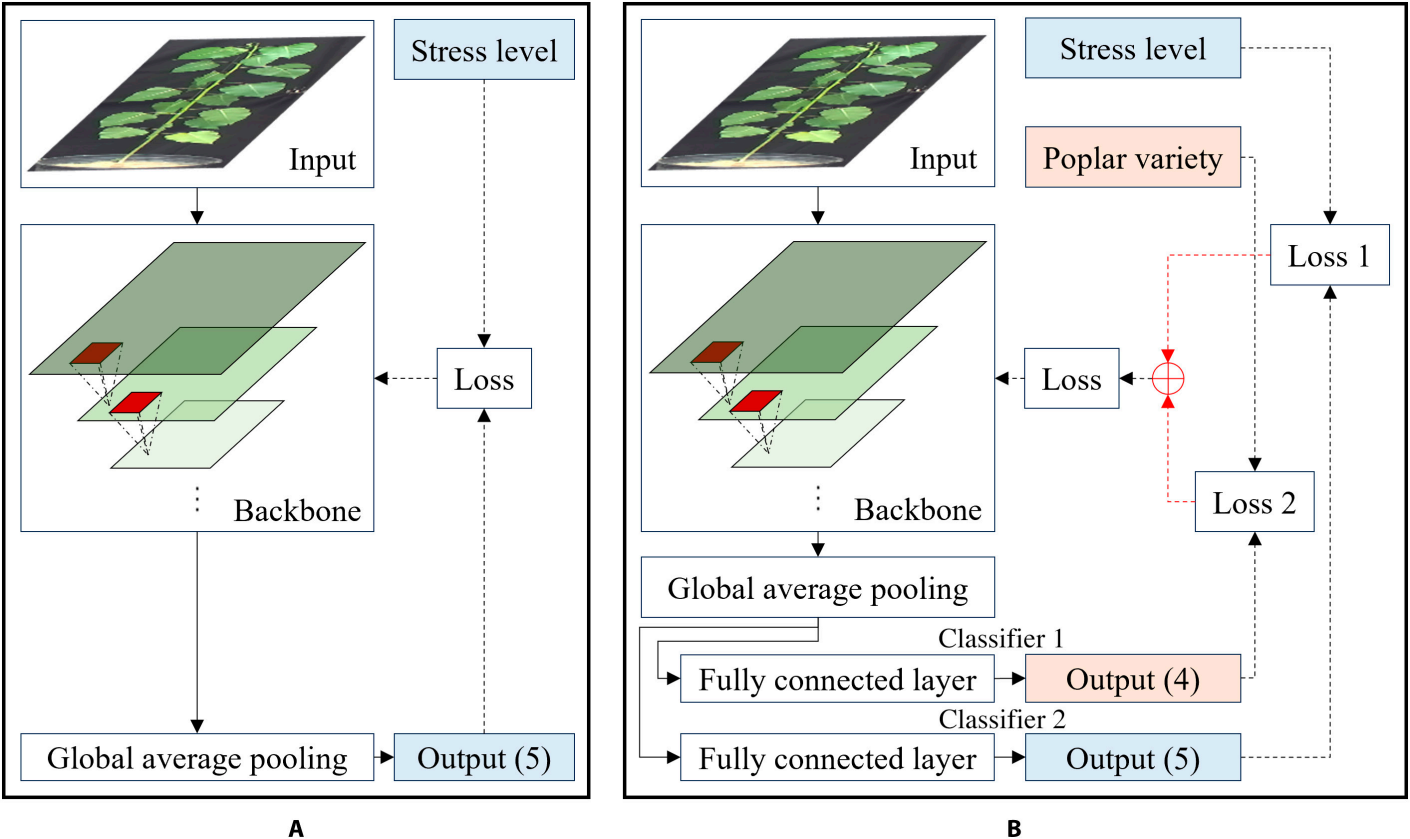
Convolutional neural networks for poplar drought stress detection. (A) Conventional single-task learning model and (B) multitask learning model.

For the second objective, conventional single-task deep learning classification and multitask learning were used and compared in this study. Single task classification used raw plant images or the digital representation of plant structure as the input and only predicted the drought stress level of the plants. However, the multitask learning methods had multiple expected outputs. These varieties were “poplar variety” and “drought stress level” in this study. The fused loss calculated on the basis of the cross-entropy losses of stress level classification and poplar variety classification were adopted for tuning the weights and bias of the convolutional neural network (CNN) models. Using such a supervised training mode, the CNN models were expected to extract deep features rich in both stress-level-related and variety-related knowledge. The designed single-task and multitask deep learning models are shown in Fig. 2.

The details of the classification models used are introduced below. The input was RGB images of a single poplar plant. For the backbone of classification models, the popular ResNet [38] and MobileNet [39] models were used and compared. In the single-task classification models, the output layer (considered a feature classifier) was a simple linear mapping unit. The output dimension of the backbone was 1,024. The loss function was the SoftMax cross-entropy loss. In the multitask classification models, there were 2 output channels. Each of them was a fully connected neural network with one hidden layer. The fused loss of 2 classification tasks was the final loss function for the multitask learning models. There were 64 neurons inside the hidden layer. The output dimension of the backbone was also 1,024, the same as that of the studied single-task learning models.

The same training configuration was used to train all the mentioned classification models. For the 576 captured images of poplar saplings, the samples of each variety were divided into training, validation, and prediction sets at a ratio of 6:2:2. The backbone was pretrained on the basis of the ImageNet dataset. The initial learning rate was 0.0005 (decreased to 1/10 after 20 epochs). The number of training epochs was set to 60 according to the trial-and-error-based experiment. The batch size was 16. During the training procedure, the model with the highest validation accuracy was saved as the best model. All the programs for deep learning were developed using Python 3.9 and the PyTorch framework.

### Digital representation of leaf growth posture

In this study, the horizontal angles of the petiole (*α*) and that of the midvein (*β*) were calculated to describe the posture of the leaves, which were highly correlated with the water status of the poplar saplings. This procedure had to be performed on the basis of the instance segmentation results. First, a group of segmented regions of the petiole and midvein from the same leaf had to be retrieved, which could be achieved by (a) selecting a segmented leaf region, (b) finding a petiole region that had the highest value of intersection over union with the current leaf region, and (c) finding the corresponding midvein in the same way.

**Fig. 3.**
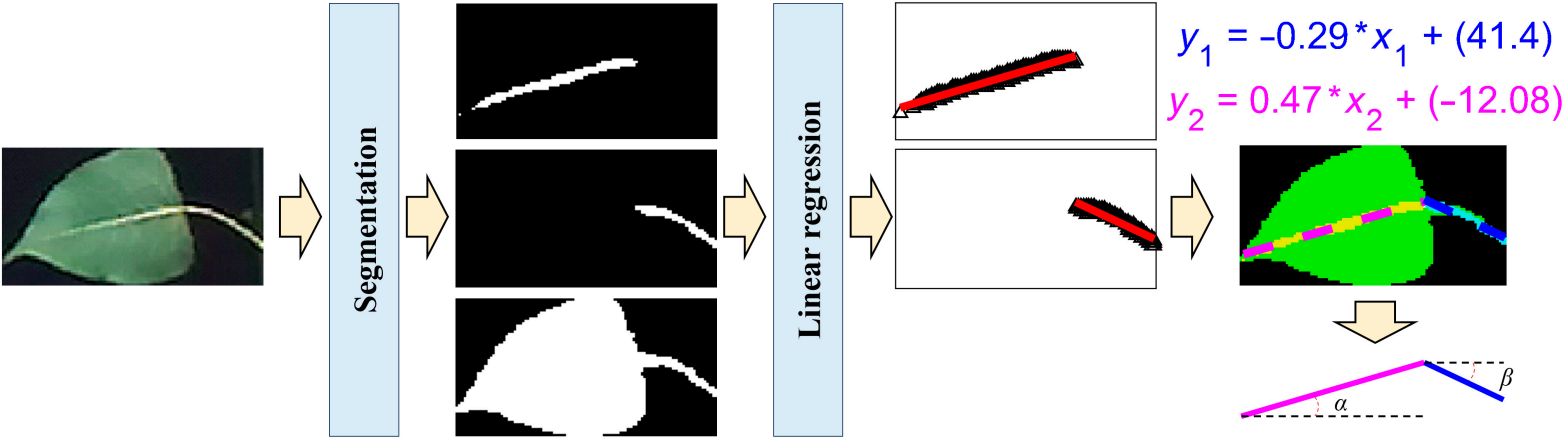
The designed method for leaf growth posture calculation.

The horizontal angle of the petiole could be calculated by the following steps: (a) The pixels covered by the petiole of the target leaf were extracted. (b) The best fitting straight line (*y* = *kx* + *b*) for the mentioned pixel points was established using a linear regression algorithm. (c) The horizontal angle of the fitted line was calculated using Arctangent(*k*). These steps are also illustrated in Fig. 3.

## Results

### Results of instance segmentation and leaf posture digitalization

**Fig. 4.**
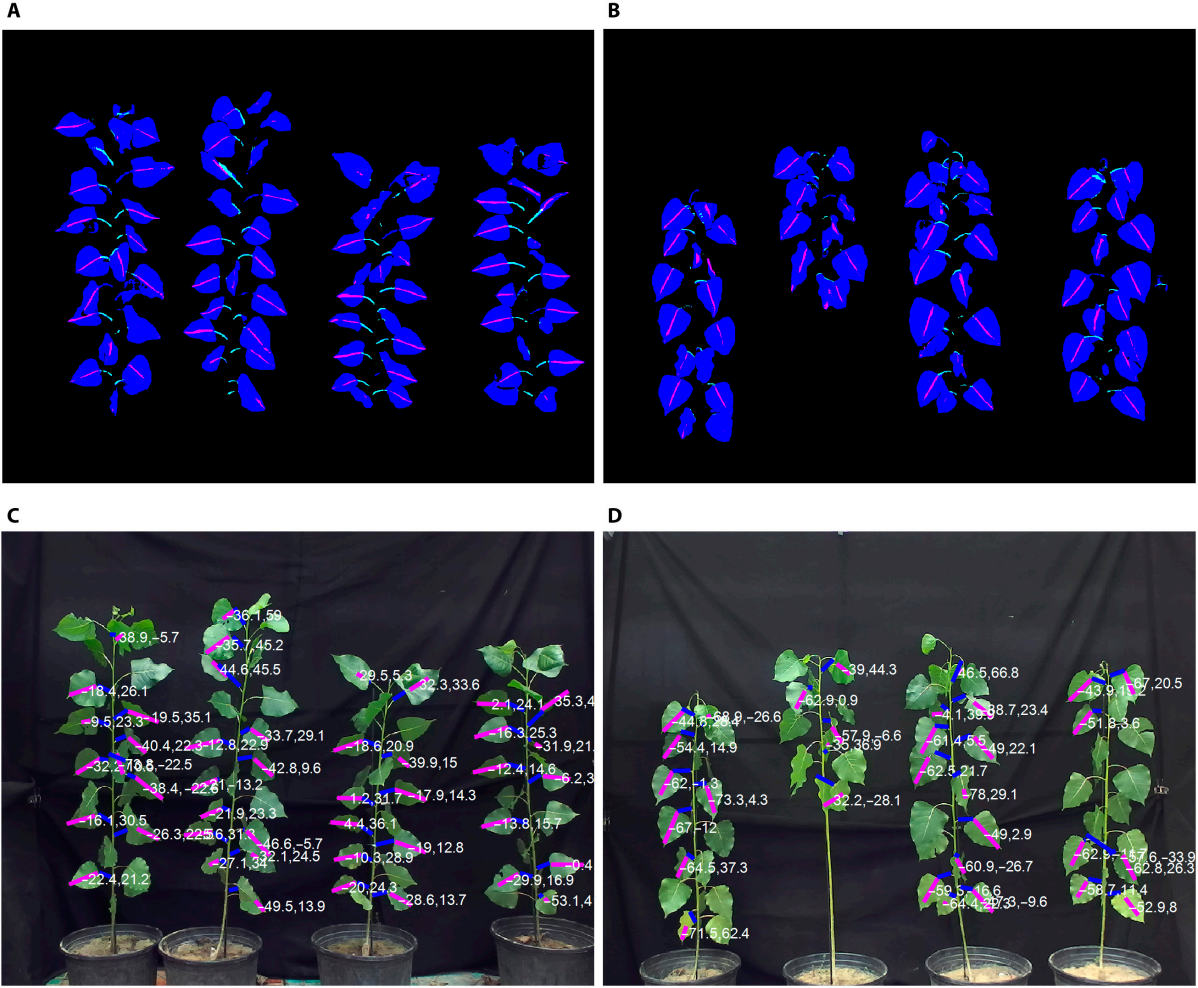
Poplar leaf growth posture calculation results. (A) and (C) are the segmentation results and the calculated leaf growth posture of samples in the CK group, and (B) and (D) are those of samples in the MD group.

Two images involving 4 healthy poplar saplings and another 4 stressed saplings were selected as examples. Figure 4 displays some results of poplar leaf segmentation and single leaf posture estimation. Figure 4A and B shows that the leaf, midvein, and petiole regions were segmented from the background. The data output from the MaskRCNN and from the YOLOv8 segmentation both separated each individual leaf by assigning different indices, although the colors of the segmented leaves are all “blue” in Fig. 4. The corresponding performance metrics are listed in Table 2. The AP_0.5_ values were used to select a better model for subsequent steps. The final performance was evaluated by the error of the angle calculation. The results showed that the FasterRCNN models performed slightly better than the YOLO models for leaf segmentation, while the YOLO models performed slightly better for detecting the midvein and petiole. The overall performances of these models were similar. Therefore, the subsequent steps for poplar leaf angle calculation were conducted on the basis of the FasterRCNN model. A potential solution for improving segmentation accuracy could be explored in future studies.

**Table 2.** Performances of MaskRCNN and YOLOv8 segmentation

Model	Target	AP_0.5_^a^					
		Training		Validation		Prediction	
		Box	Segmentation	Box	Segmentation	Box	Segmentation
YoloV8-seg	Leaf	0.908	0.819	0.892	0.806	0.479	0.589
	Midvein	0.692	0.336	0.701	0.332	0.643	0.333
	Petiole	0.762	0.49	0.767	0.447	0.77	0.354
MaskRCNN	Leaf	0.949	0.905	0.949	0.905	0.438	0.633
	Midvein	0.803	0.179	0.775	0.154	0.676	0.12
	Petiole	0.766	0.689	0.766	0.689	0.542	0.475

^a^“Box” indicates that the AP_0.5_ values were calculated for the bounding boxes predicted by the models. “Segmentation” indicates that the AP_0.5_ values were calculated for the masks predicted by the models.

On the basis of the method described in the “Digital representation of leaf growth posture” section, the fixed lines of the petiole and midvein of each leaf could be found. Then, the angles *α* and *β,* which indicate the leaf growth posture, were calculated. It should be noted that some errors could be detected before leaf angle calculation. Inside a selected segmented leaf region, the segmented midvein region covering less than a certain number of pixels had to be moved, which was not enough to conduct linear regression. Moreover, segmented leaf regions without effective midvein or petiole areas had to be considered invalid leaf individuals. Therefore, in Fig. 4C and D, there are some leaves not annotated with lines and angle values.

The accuracy of the leaf angle calculation was further evaluated. A new group of captured poplar sapling images was annotated manually (10 images not used in the model training procedure; a total of 40 plants were processed), defined as the *D*_angle test_. The MaskRCNN model trained only on the simulated dataset was used to process the new *D*_angle test_ dataset. Then, the values of leaf angle calculated on the basis of the manual annotation and those based on the segmentation output were compared. The mean absolute error (MAE) was used as the performance indicator.

**Fig. 5.**
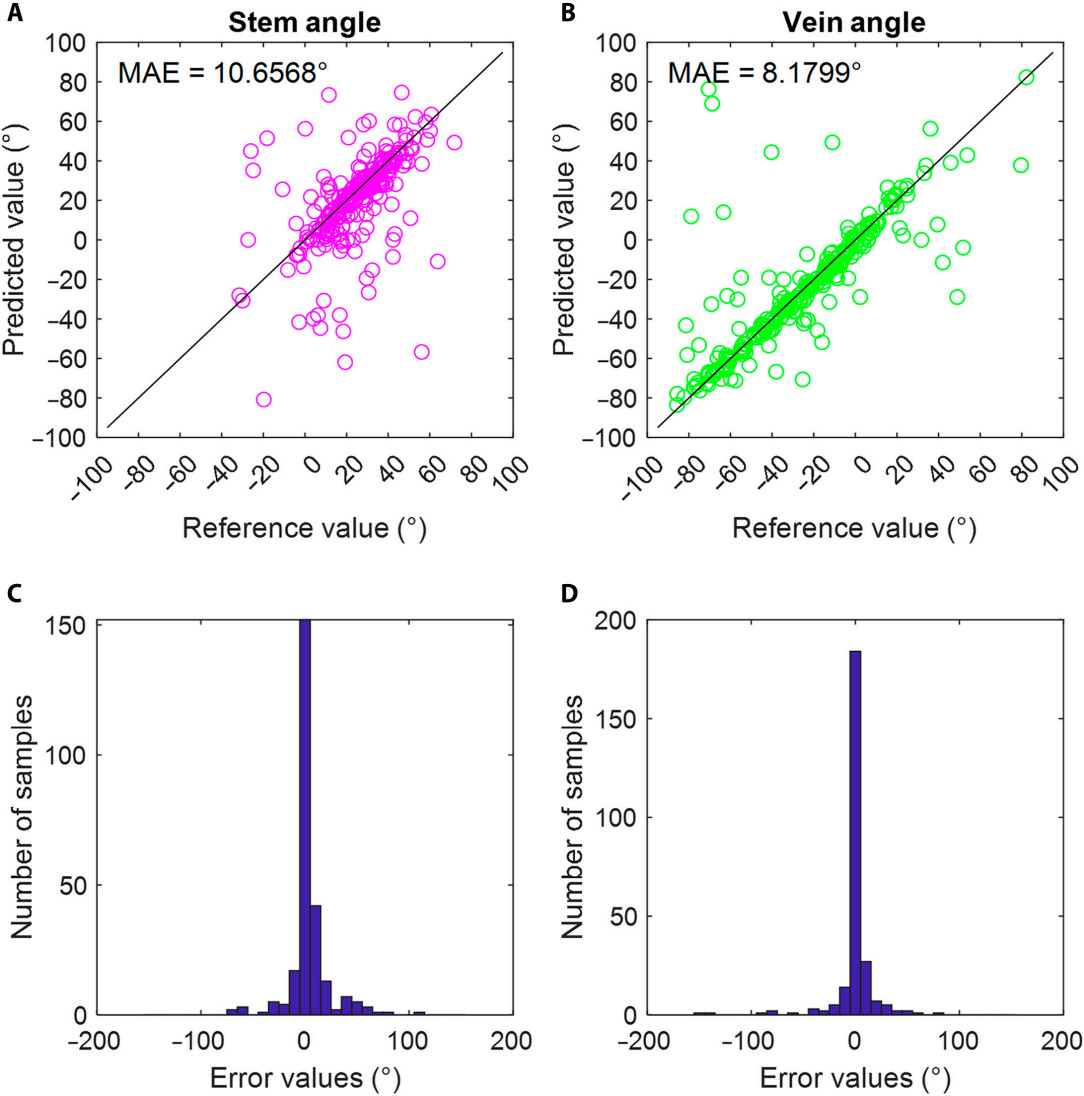
Leaf posture calculation results. (A) Petiole angle calculation result, (B) midvein angle calculation result, (C) distribution of petiole angle calculation errors, and (D) distribution of midvein angle calculation errors.

In total, 256 complete leaves were segmented from the *D*_angle test_ dataset. Figure 5A and B shows that the MAEs of the petiole angle calculation and midvein angle calculation were approximately 10.7° and 8.2°, respectively. Most of the scatter points were located near the line *y* = *x*. Some of the points far from the *y* = *x* line increased the MAE values. Figure 5C and D shows the frequency distribution histograms of the error of the petiole angle calculation and midvein angle calculation. Approximately 70% of the error values were within a range of −5° to +5°. There were very few points with errors greater than 50°, which could be attributed to incomplete segmentation of the target areas (leaf, petiole, and midvein). In particular, for processing SD-stressed samples, the petiole exhibited a parabolic shape. Fitting with the linear regression method resulted in marked angle deviation when the petiole was not fully segmented. Afterward, the deep learning segmentation model was trained using a simulated dataset generated from very few manually annotated images; therefore, these results are promising.

**Fig. 6.**
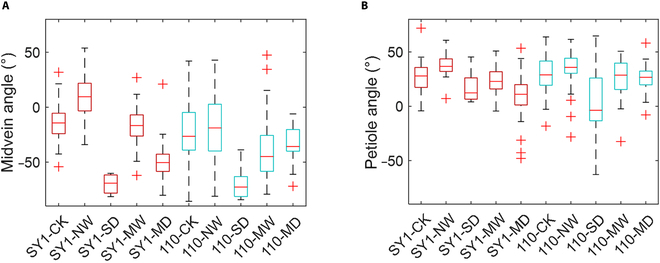
Statistical results of the horizontal inclination angles of different leaves for SY1 and 110. (A) Angle of the midvein and (B) angle of the petiole.

Then, the calculated leaf posture information of the different groups of plants was further analyzed. Figure 6 shows the statistics of SY1 and 110 for comparison and discussion. The plants in the SD stress group exhibited the smallest midvein angle and petiole angle among the 5 groups. After the plants were subjected to drought stress, the amount of water needed for rewatering increased at both angles. These results were consistent with the fact that drought caused wilting of the leaves and that reirrigation could restore leaf conditions. By comparing Fig. 6A and B, the impact of drought stress on the horizontal inclination angle of the midvein was greater than that on the horizontal inclination angle of the petiole. Therefore, the leaf posture calculation method was useful and valuable for plant status analysis.

### Poplar drought stress grading results based on image classification

The results of poplar plant drought stress detection using image classification are discussed in this section. Three kinds of modeling strategies were used. In addition, 2 CNN backbones (MobileNet and ResNet) and 2 kinds of modeling strategies (single-task learning and multitask learning) were compared. The results are listed in Table 3.

• Modeling method A. Modeling without considering the differences in plant variety. One variety of plant was selected for prediction. For example, SY1 was used for prediction, and samples of the remaining 3 varieties (3804, 895, and 110) were divided 3:1 for training and validation for training the MobileNet model. This model was defined as MobileNet-1. The ResNet model using samples of the “895” variety as the prediction dataset was defined as ResNet-3. The corresponding results are shown in the first to eighth rows in Table 3.
• Modeling method B. Single-task learning-based modeling that considers differences in plant variety. The samples of each variety were divided into training, validation, and prediction sets at a ratio of 6:2:2, as described in the “Image classification” section. The corresponding results can be seen in the 9th and 10th rows in Table 3.
• Modeling method C. Multitask-learning-based modeling that considers the impact of different varieties on drought stress grading. The same dataset configuration used for single-task learning-based modeling was used. The models were expected to simultaneously predict the poplar variety and drought stress level; see the results in the 11th and 12th rows in Table 3.

**Table 3.** Performances of the deep learning models on the training, validation, and prediction datasets

Model ^a^	Task	Classification accuracy (%)		
		Training	Validation	Prediction
MobileNet-1	Stress degree	78.52	70.99	39.58
MobileNet-2	Stress degree	83.70	61.11	50.00
MobileNet-3	Stress degree	95.19	67.28	62.50
MobileNet-4	Stress degree	95.93	74.07	62.50
ResNet-1	Stress degree	81.11	70.37	43.06
ResNet-2	Stress degree	85.19	64.20	50.69
ResNet-3	Stress degree	95.56	70.99	64.58
ResNet-4	Stress degree	92.96	69.14	56.94
S-MobileNet	Stress degree	100	70.04	68.52
	Variety	–	–	–
S-ResNet	Stress degree	100	72.01	68.52
	Variety	–	–	–
M-MobileNet	Stress degree	100	75.93	75.93
	Variety	100	97.22	99.07
M-ResNet	Stress degree	100	75.00	73.15
	Variety	100	100	99.07

^a^MobileNet-1 to MobileNet-4 and ResNet-1 to ResNet-4 represent the models trained with different sources. The suffix “-1” indicates that the first variety was for prediction, and the remaining 3 varieties were for training and validation. The same is true for the other suffixes. “S” and “M” denote single-task learning and multitask learning, respectively.

First, modeling method A was analyzed. The first to eighth rows in Table 3 show that the accuracy values on the training dataset were good. However, lower accuracies were found for the validation and prediction sets. The prediction accuracies were all lower than 65%. These findings indicated that different varieties of poplar might respond differently to the same level of drought stress. This factor must be considered in modeling.

**Fig. 7.**
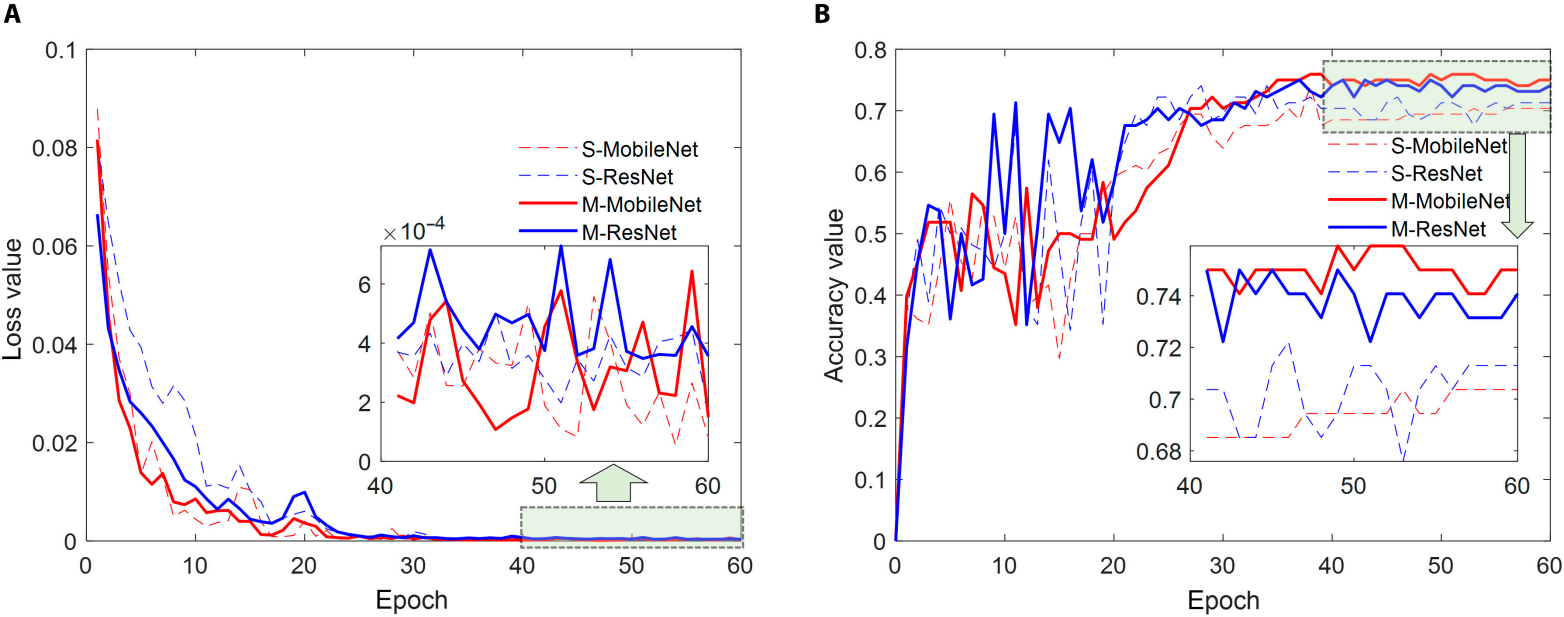
Comparison of modeling performances between different CNN models and different modeling strategies. (A) Training loss values and (B) stress grading accuracy values on the validation dataset. “S” denotes single-task learning, and “M” denotes multitask learning.

Then, modeling methods B and C were examined in detail; see the 9th and 12th rows in Table 3. The curves of training loss (cross-entropy loss for stress degree classification) and validation accuracy of the studied models are plotted in Fig. 7. Figure 7A shows that as the number of iterations increased, the loss values of all 4 compared models gradually converged. After training for 60 epochs, the training losses of these models decreased to values lower than 7 × 10^−4^. In Fig. 7B, the corresponding validation accuracies of each epoch are plotted. From epochs 1 to 20, the accuracy values showed a rapid oscillating upward trend. During subsequent cycles, these values slowly increased and tended to stabilize. Within a range of 40 to 60 epochs, the accuracy curve of the multitask learning MobileNet was greater than that of the single-task learning MobileNet (a comparison between the solid curves and dashed curves). A similar situation could be observed for the ResNet models.

During model training procedures, the model (more specifically, a group of parameters) that achieved the highest validation accuracy was saved. Then, the optimized models were evaluated on training, validation, and prediction datasets. For the studied deep learning models trained using methods 2 and 3, the accuracies on the validation dataset were very close to those on the independent prediction dataset. Multitask learning models also outperformed single-task learning models on the individual prediction dataset, for which the improvements in prediction accuracy were greater than 5%. Therefore, it could be concluded that multitask deep learning reached higher accuracies for the studied poplar stress detection task than single-task learning methods. The selected backbone of the CNN models also influenced the modeling accuracy. In this study, M-MobileNet (accuracy = 75.93%) performed slightly better than ResNet (accuracy = 73.15%).

On the other hand, both M-MobileNet and M-ResNet achieved perfect accuracies (close to 100%) for the poplar variety discrimination task. It could be inferred that the differences between different varieties were relatively obvious; therefore, it was easy for the CNN models to extract the deep features that could represent such differences. In summary, the results shown in Table 3 prove the effectiveness and progressiveness of multitask deep learning for poplar drought stress detection.

**Fig. 8.**
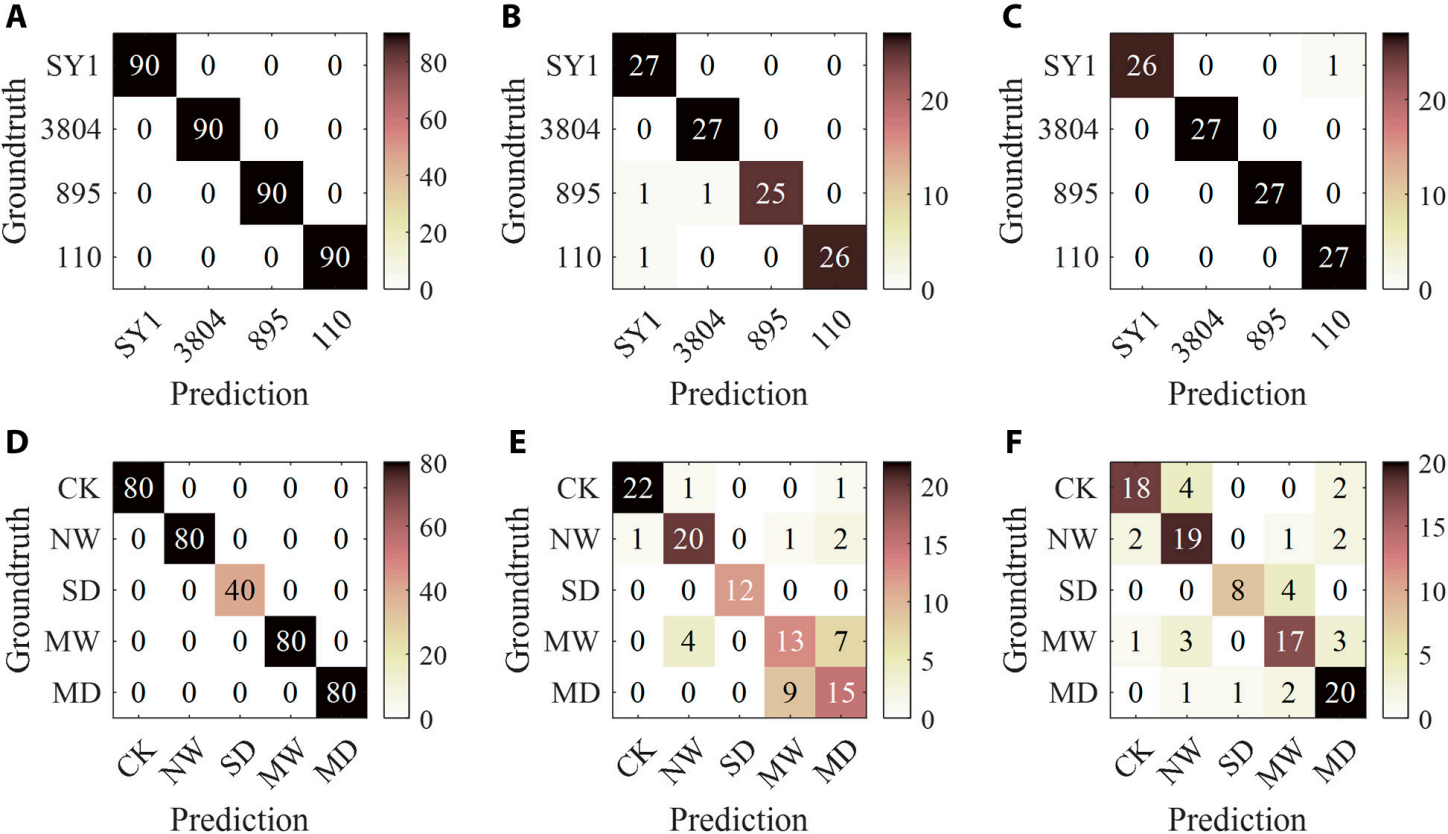
M-MobileNet confusion matrices. (A to C) Poplar variety identification results on the training, validation, and prediction datasets, respectively. (D to F) Poplar drought stress level classification results on the training, validation, and prediction datasets, respectively.

Because of the similarities between the poplar samples under different degrees of drought stress (e.g., the CK group and the NW group), the difficulty of discriminating drought stress levels was relatively high. The highest prediction accuracy, 75.93%, was realized by M-MobileNet. Figure 8 shows the confusion matrices of the best model for further analysis. The highest error classification rate could be observed for the “MW” stress level on both the validation dataset and prediction dataset. The lowest error rate was located in the “SD” row. Hence, the distribution of error classifications on the validation dataset was similar to that on the prediction dataset, which illustrated the stability of the trained model.

## Discussion

In this study, 2 tasks were carried out for the phenotyping of drought-stressed poplar saplings. The first task involved determining the poplar leaf posture. There have been several published deep-learning-based human posture detection algorithms [15,40]. Key points included the head, shoulder, elbow, wrist, knee, foot, fingertip, and so on. For human posture analysis, the quantity of these points is fixed. However, when processing the posture estimation of poplar plants, the number of leaves is variable. New solutions should be studied for processing plant samples. For plants with simple structures, such as maize, posture can be quickly described by a skeletonization algorithm [25], which can be conducted using existing image processing tools (e.g., MATLAB). To calculate the leaf angle in a more precise way, the leaf collar can first be detected as an ROI. Then, key point detection can be performed inside this extracted ROI. The form of poplar leaves differs from that of maize leaves, which are very difficult to digitalize via a skeleton extraction algorithm. Hence, the key components of poplar plants, such as single leaves, should first be separated from the whole plant. Then, the parameters of a single leaf can be calculated. Next, the statistical results of the leaf parameters can be used to describe the status of one plant or a group.

Commonly, leaf instance segmentation model training requires a large number of manual annotations [41,42], and there is a major shortage of such methods. Although popular generative adversarial networks can generate realistic plant images [31], groundtruth mask preparation for leaf segmentation still requires much manual work. Such methods are more suitable for generating plant images for classification tasks. Exemplar-based data generation methods were studied to realize dataset augmentation, utilizing touching seed segmentation [43] and leaf counting [44] tasks. A small batch of plant images were annotated and used to construct the component pool (e.g., leaf image pool and seed image pool). A large number of synthetic image–annotation pairs could be rapidly generated by combining these annotated components. However, in these mentioned cases, the components (leaves and seed kernels) were randomly arranged in the background images, which might make the “pattern” of synthetic images different from that of real images, leading to missing detection issues. In addition, complete codes or programs for synthetic image–annotation pairs were rarely provided in the existing articles. In this study, a small number of annotated plant leaves and plant trunks were used as the basic components to generate a large number of simulated poplar sapling images for model training. All the leaves were “mounted” on the plant trunk, promoting the pattern of synthesized images to be closer to that of real ones. The proposed image and annotation generation method reduced the manual workload for labeling, which can also be regarded as a few-shot learning strategy [45]. The complete codes for dataset augmentation were published. The published program can output annotated files of different formats required by the most popular instance segmentation networks, including MaskRCNN and YOLOv8-seg. In future studies, the proposed methods could be further used for synthesizing images and groundtruth values of other objects, such as fruit trees densely covered with fruits, leaves covered with disease spots, and edible fungi growing on substrates, assisting in fruit picking [46], disease identification [32], and mushroom production estimation [47], respectively.

The second task was to assess the drought stress level of the poplar saplings. Some studies have used one variety of plant for drought level classification or other plant stress detection [48,49]. Although a good performance was achieved, this kind of method might not be applicable for stress detection in multiple plant varieties. Different varieties of plants have different stress tolerances. For instance, when plants suffer from the same level of drought stress, their degree of change in phenotypic information varies. If a deep learning model was applied for irrigation level classification of different varieties of plants, the model might learn the phenotypic features originating from variety differences rather than drought-stress-related features. In similar studies, it was found that the variety of plants had a certain impact on nutrient content inspection and disease detection tasks [50]. Hence, variety-related responses should be considered when performing plant stress detection tasks. Multitask learning models are trained using multiple label constraints [47], which forces the models to extract deep features simultaneously associated with multiple expected outputs. These models have been widely applied for multiple phenotypic trait determination using only one model [51]. However, there is a relative lack of research that uses multitask learning to reduce the impact of plant variety on plant drought stress grading. In this study, multitask learning was adopted for simultaneously predicting poplar variety and drought level. The subnetworks for variety classification and drought grading shared the same feature map, which included both variety-related and drought-related information. Therefore, the multitask learning model performed better than conventional single-task deep learning models. The methods proposed in this study have great potential for drought-resistant poplar sapling screening and for precise irrigation of poplar samples with different drought tolerances.

**Fig. 9.**
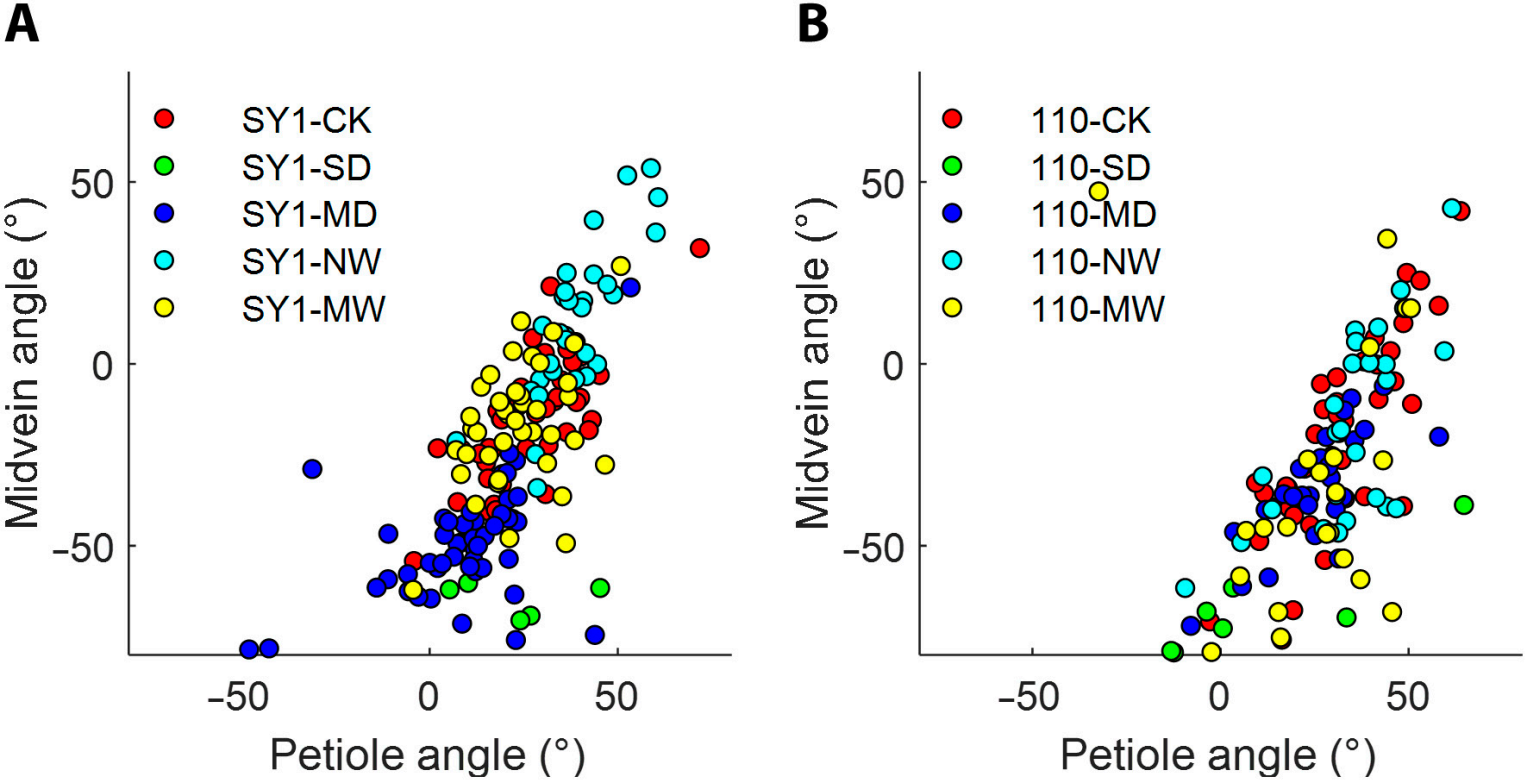
Scatter plots of midvein angles and petiole angles of different poplar varieties under different levels of drought (SY1 and 110). (A) SY1 and (B) 110.

The impacts of drought on the midvein angle and petiole angle were also evaluated. In Fig. 9, the midvein angle and petiole angle were considered 2 characteristics for visualizing the differences between samples under different stress levels. Overall, the scatter plots representing the same group of samples tended to cluster together. When considering only the CK, SD, and MD groups, these 3 groups were relatively easy to separate. The results indicated that obvious differences in leaf angles (midvein and petiole) existed between these 3 groups. Considering only the other 2 groups (NW and MW groups), different frequencies of rewatering had a certain impact on leaf posture. However, the leaf posture of the samples from the NW and MW groups was similar to that of the CK group. The leaf angles of the plants subjected to SD followed by NW were greater than those of the plants in the CK group. Therefore, based only on the angles of the midvein and petiole, it was still difficult to separate the samples under the studied 5 drought stress levels. Nevertheless, leaf posture is an important drought-related phenotype. Future studies can be conducted using the time-series sequence of posture information [52]. The inspection of dynamic changes in plant posture might be a promising approach for accurately grading drought stress.

There were also some limitations in this study, which can be addressed in future studies. First, the ZED Mini 2 camera used was a binocular camera that could provide both color images and the corresponding 3-dimensional cloud points. Only the color images were processed in this study to establish a low-cost phenotyping method. In future research, the 3-dimensional posture of plants can be analyzed to better understand the growth status of plants. Other water content-sensitive phenotypes, such as leaf wilting [52], should also be considered to improve drought stress detection methods. Moreover, limited samples of poplar plants were studied. More samples should be cultivated, observed, and analyzed in the future to further improve the robustness of the deep learning models and identify more reliable patterns of phenotypic changes in poplar plants originating from drought stress.

## Conclusion

This paper proposes new methods for drought-stressed poplar sapling phenotyping and drought stress level determination. The leaf posture analysis task incorporates 2 unique techniques. The first part is synthesizing a training dataset using a combination of plant organ segmentation. The image–annotation pairs are automatically generated, markedly reducing manual labeling. The second part is plant-structure-based analysis, which evaluates the angle of leaves obtained by segmenting leaves and the corresponding stems. Promising results showed that the MAEs of the angle calculations were 10.7° and 8.2° for the per-leaf estimations of the petiole and midvein, respectively. With the published codes for dataset augmentation, the proposed plant image–annotation pair synthesis method can be transferred quickly to other areas. For instance, disease spots and fruits can be generated for diseased leaf segmentation and vision-based fruit detection and picking, respectively. In addition, the multitask deep learning classification algorithm can be utilized to improve the drought stress level classification performance. The model was trained using both variety information and stress level information as supervision constraints, producing deep features that simultaneously revealed the stress level and poplar variety. Considering the impact of poplar variety, the multitask-learning-based models outperformed single-task learning models, reaching the highest accuracy, 99% for variety discrimination and 76% for stress level grading. The proposed phenotyping methods can benefit applications such as drought-stress-resistant plant screening and irrigation decision-making in regard to broader kinds of plants.

## Data Availability

The codes for conducting the proposed poplar plant image generation method and for annotation format conversion were uploaded to the GitHub platform (https://github.com/L-Zhou17/Plant-Image-Generation). Other codes and datasets are available upon request.
